# On the remarkable nonlinear optical properties of natural tomato lycopene

**DOI:** 10.1038/s41598-022-12196-3

**Published:** 2022-05-31

**Authors:** N. Numan, S. Jeyaram, K. Kaviyarasu, P. Neethling, J. Sackey, C. L. Kotsedi, M. Akbari, R. Morad, P. Mthunzi-Kufa, B. Sahraoui, M. Maaza

**Affiliations:** 1 UNESCO-UNISA-iTLABS/NRF Africa Chair in Nano-Sciences & Nanotechnology, CGS, University of South Africa, Muckleneuk ridge, Pretoria, 0001 South Africa; 2 NANOAFNET, iThemba LABS-National Research Foundation of South Africa, 1 Old Faure Road, Cape Town, 7129 Western Cape South Africa; 3 Department of Physics, School of Engineering and Technology, Surya Group of Institutions, Vikravandi, Villupuram, Tamilnadu 605652 India; 4 Physics Department, Laser Research Institute, Stellenbosch University, Stellenbosch, Western Cape South Africa; 5 LUNAM Université, Université d’Angers, 2 Bd Lavoisier, 49045 Angers Cedex, France; 6 CNRS UMR 6200, Laboratoire MOLTECH-Anjou, 2 Bd Lavoisier, 49045 Angers Cedex, France; 7 National Laser Centre, Council for Scintific & Industrial Research, Meiring Naude road, Pretoria, 0001 South Africa

**Keywords:** Biophysics, Nanoscience and technology, Optics and photonics, Physics

## Abstract

In line with the renewed interest in developing novel Non Linear Optical (NLO) materials, natural Lycopene’s NLO Properties are reported for the first time within the scientific literature. Correlated to its 1-D conjugated π-electrons linear conformation, it is shown that natural Lycopene exhibits a significantly elevated 3rd order nonlinearity χ^(3)^ as high as 2.65 10^−6^ esu, the largest value of any investigated natural phyto-compound so far, including β-carotene. In addition to a saturable absorption, the corresponding observed self-defocusing effect in Lycopene seems to be the result of a thermal nonlinearity. The nonlinear response coupled to the observed fluorescence in the Visible spectral range points to a potential photodynamic therapy application as well as the possibility of engineering of novel hybrid Lycopene based NLO nano-materials.

## Introduction

Nonlinear Optical (NLO) materials have progressed gradually over the past 20 years with notwithstanding an ongoing intensive research in view of discovering new NLO materials^1–3^. Such a research of novel NLO materials is driven by the pressing ICT related technological photonics applications, particularly in logic systems, all optical switching, frequency conversion, light amplification, optical bistability, all optical switching among others. In addition, NLO materials became pivotal as per their critical role for high-speed information processing towards addressing the challenges of reduced energy consumption and enhanced speed as well as bandwidth in the various modern ICT technologies.

NLO materials exhibit a significant large second order or third order optical susceptibilities χ^(2)^ or χ^(3)^. One can distinguish inorganic and organic families. Among the inorganics, Beta Barium Borate (BaB_2_O_4_), Barium titanate (BaTiO_3_), Lithium niobate (LiNbO_3_), KH_2_PO_3_ (KDP), Lithium tantalite (LiTaO_3_) and potassium niobate (KNbO_3_) are χ^(2)^ optical switching and frequency doubling materials that have been studied for decades. Among the organics, there is a large number of χ^(3)^ and χ^(2)^ organic materials, including dyes, dimethylamino nitrostilbene, methyl nitroanaline, poly-BCMU, polydiacetylenes, and urea^4^. Because they are essentially chains, many organic molecules can be easily polarized and therefore exhibit higher order susceptibilities. The polarizability of organic materials is often enhanced by the mobility of the delocalized π-electrons in the C–C bonds in aromatic rings. Likewise, hybrid NLO materials have even been engineered by combining organic and inorganic components such as metalo-phthalocyanines which display strong excited state absorptions^5,6^.

Among the first organic NLO materials, one could single out Coumarin which was derived, initialy, from a natural compound; the Tonka bean^7^. Coumarin natural dyes family was at the origin of the first series of tunable laser sources; the dye Laser sources. Yet, Coumarin molecule is non-fluorescent, it displays intense fluorescence properties upon the substitution of functional groups at different positions. In addition to Coumarin, several natural compounds were found to exhibit a significant NLO response in the VIS & IR spectral range including natural Chlorophyl^8–11^.

**Figure 1:**
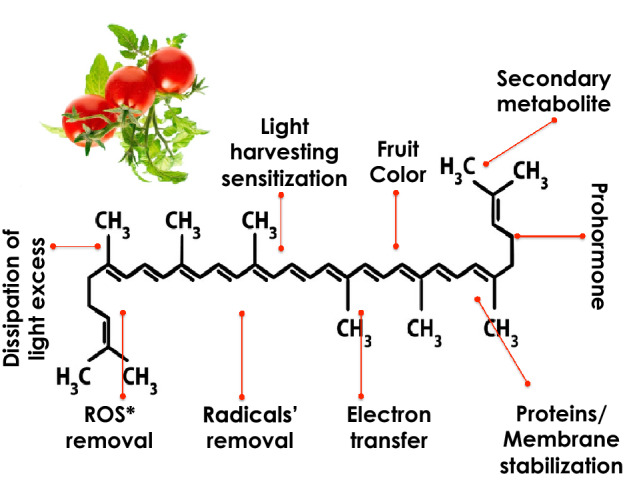
1-D Chemical structure of p-conjugated electrons of Lycopene and its various functionalities.

As mentioned previously, it was shown that the conjugated quasi 1-D π-electron systems, such as semiconducting polymers exhibit an enhanced third-order NLO response. Accordingly, and as Lycopene extracted from tomato fruit, possesses such a 1-D π-electrons electronic conformation (Fig. 1), it should exhibit a χ^(3)^ response too. Indeed, Lycopene from tomato natural extract is a terpene consisting of 8 isoprene molecules with a chemical formulation of C_40_H_56_. Lycopene from tomato Is a π-electrons conjugated carbon chain long molecule (Fig. 1). As in the case of the large carotenoids family, the backbone of such a molecule consists of alternating carbon single and double bonds^12^. More precisely, Lycopene has 11 conjugated carbon double bonds along its backbone, two un-conjugated double bonds, and no end groups (Fig. 1).

In addition of tomatoes as a source of Lycopene, this latter is found in several photosynthetic pigment-protein complexes in plants (Watermellon, Wolfberry, Papaya, Seabuckthorn, ….), photosynthetic bacteria (Saccharomyces cerevisiae, …), fungi, and algae^13–15^. They are responsible for the bright red–orange color of fruits and vegetables, and perform various functions (Fig. 1) in photosynthesis, and protect photosynthetic organisms from excessive light damage. Lycopene is a key intermediate in the biosynthesis of carotenoids including b-carotene, and xanthophylls.

Likewise, and from a medical perspective, Lycopene was found to exhibit a significant activity as membrane-protective antioxidants which efficiently scavenge ^1^O_2_ and trap peroxyl radicals (ROO*)^16–19^. In addition, anticancer, antiproliferative and pro-differentiation activities have been attributed to Lycopene^20,21^. This set of medicinal properties makes of Lycopene, an appropriate carotenoid to be investigated from nonlinear optical perspective. Furthermore, its chemical and thermal stability within the broad family of carotenoids makes it ideal as a potential NLO in thin films structure, if any.

Henceforth, the novelty of this contribution lies within the fact that it reports for the first time in the scientific literature the NLO responses of Lycopene. It is hoped that the obtained results could firstly, trigger a broader interest within the photonics community and, secondly, be exploited for potential photodynamic therapy or/and skin cancer treatment of lycopene based green photosynthetizer specifically as well as engineering novel NLO Lycopene based materials in view of its chemical and thermal stabilities.

## Experiments, results and discussion

### Materials and methods and lycopene extraction

Fresh ripe tomatoes identified as Lycopersicon esculentum (Solanaceae), required for the extraction of lycopene were purchased from a local market. After washing, the raw tomatoes were cleaned, homogenized, and stored at 9 °C in a glass bottle until analysis. Before the final sample preparation, the tomato fruits were immersed in boiling water for 2–3 min. The paste of tomato was prepared by a mixer crushing and 100 gm of it was placed in a 250 ml beaker. The sample was submitted to a filtration phase through Whatman n^o^1 and n^o^42 filter papers. Lycopene was extracted using a sample of ethanol (1:1, v/v), and quantified spectrophotometrically at 472 nm and expressed accordingly in mg/100 g FW according to the established procedure by Periago et al., Fish et al., and Lavecchia et al.^22–24^. Additional solvents were used including acetone, hexane, cyclohexane, and ethyl acetate (purchased from E-Merck (99.9%) were used as solvents. Based on their molecular dipoles, the various solvents were tested in view of identifying those extracting the maximum of lycopene. All the chemicals used in the study were of analytical grade. For the various expriments, solution of lycopene dissolved in hexane (0.1 mg of Lycopene in 100 ml hexane) were used.

### Sample characterizations

The UV–VIS Absorbance and the fluorescence spectra were acquired using Ocean Optics units within the spectral range of interest of 250–800 nm. The fluorescence measurements were recorded using a fibre-optics linked Ocean Optics system consisting of a UV light-emitting diode source coupled to a high sensitivity QE Pro-FL spectrometer. The excitation wavelength was fixed at 240 nm. The Raman spectroscopy investigations were carried out on a Horiba LABRAM unit with a laser green excitation of 514.5 nm.

### Z-scan experiments

**Figure 2:**
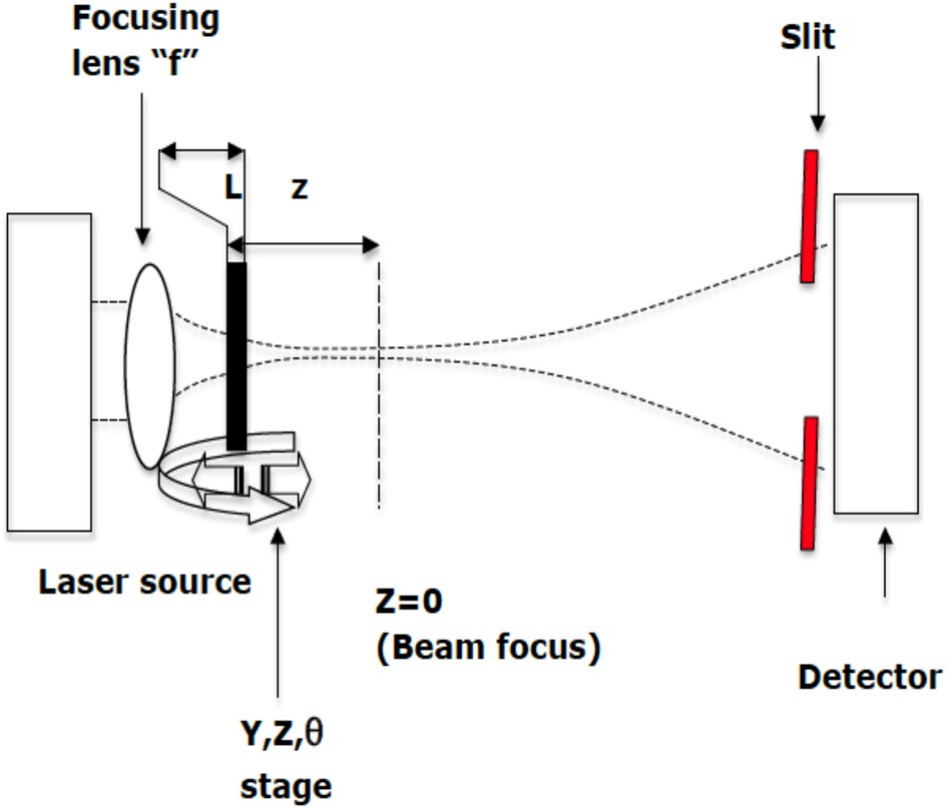
Schematic configuration of the Z–scan experimental setup used in the present study. The experimental setup consists of a CW laser source operating at 650 nm wavelength with a total power of 5 mW.

Figure 2 depicts the Z–scan experimental setup used in the present study. It consisted of a CW laser source operating at 650 nm wavelength with total power of 5 mW. The nonlinear index of refraction, n_2_ and nonlinear coefficient of absorption, β of lycopene were measured by closed and open aperture Z–scan method respectively. In the closed aperture configuration, the aperture was placed before the detector while in the open aperture geometry; a convex lens was used to collect the beam. A convex lens with focal length of f = 5 cm was used to focus the beam on to the sample. The cuvette of thickness 1 mm in thickness was mounted on a translational stage and moved the sample in the Z directions within the − 20– + 20 mm range. The transmittance of the beam was measured by a photodetector fed to a digital power meter. The thin sample approximation was used as the measured Rayleigh length was greater than the sample’s length.

### Linear optical investigations

**Figure 3:**
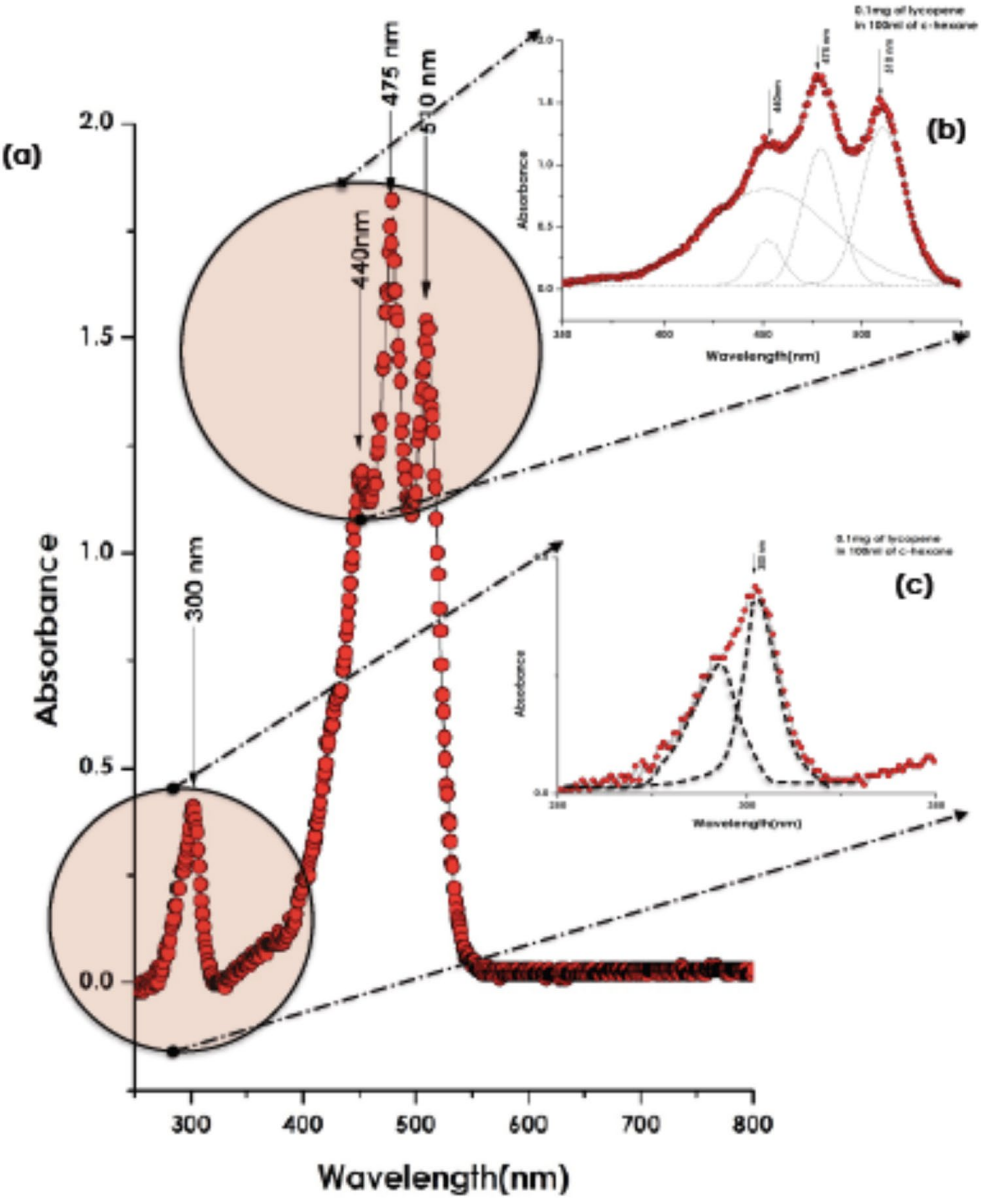
(**a**) Absorbance spectrum within the spectral range of 250–800 nm for a solution of lycopene dissolved in hexane (0.1 mg of Lycopene in 100 ml hexane), (**b**) Zoom on the major Absorbance peak, (**c**) Zoom on the deep UV Absorbance.

Figure 3a reports the absorption spectrum within the spectral range of 250–800 nm for a solution of lycopene dissolved in hexane (0.1 mg of Lycopene in 100 ml hexane). In addition to a low intensity peak located in the UV region at about ~ 300 nm, one observes 3 strong absorbance peaks centered at 440, 475, and, 510 nm respectively (Fig. 3b). These lycopene intrinsic electronic absorptions, relatively intense, are due to the various π–π* and σ–σ* transitions. The ~ 300 nm absorbance peak which is, a priori, a convolution of 2 peaks (Fig. 3c), is likely to be caused by an aggregation of the pigment molecules with participation of the solvent molecules as suggested by Hager^25,26^. This formation of polymers-like would lead to an alteration in the distribution of the electrons in the chromophore system of the lycopene molecule and thereby to a potential change of the light absorption.

**Figure 4:**
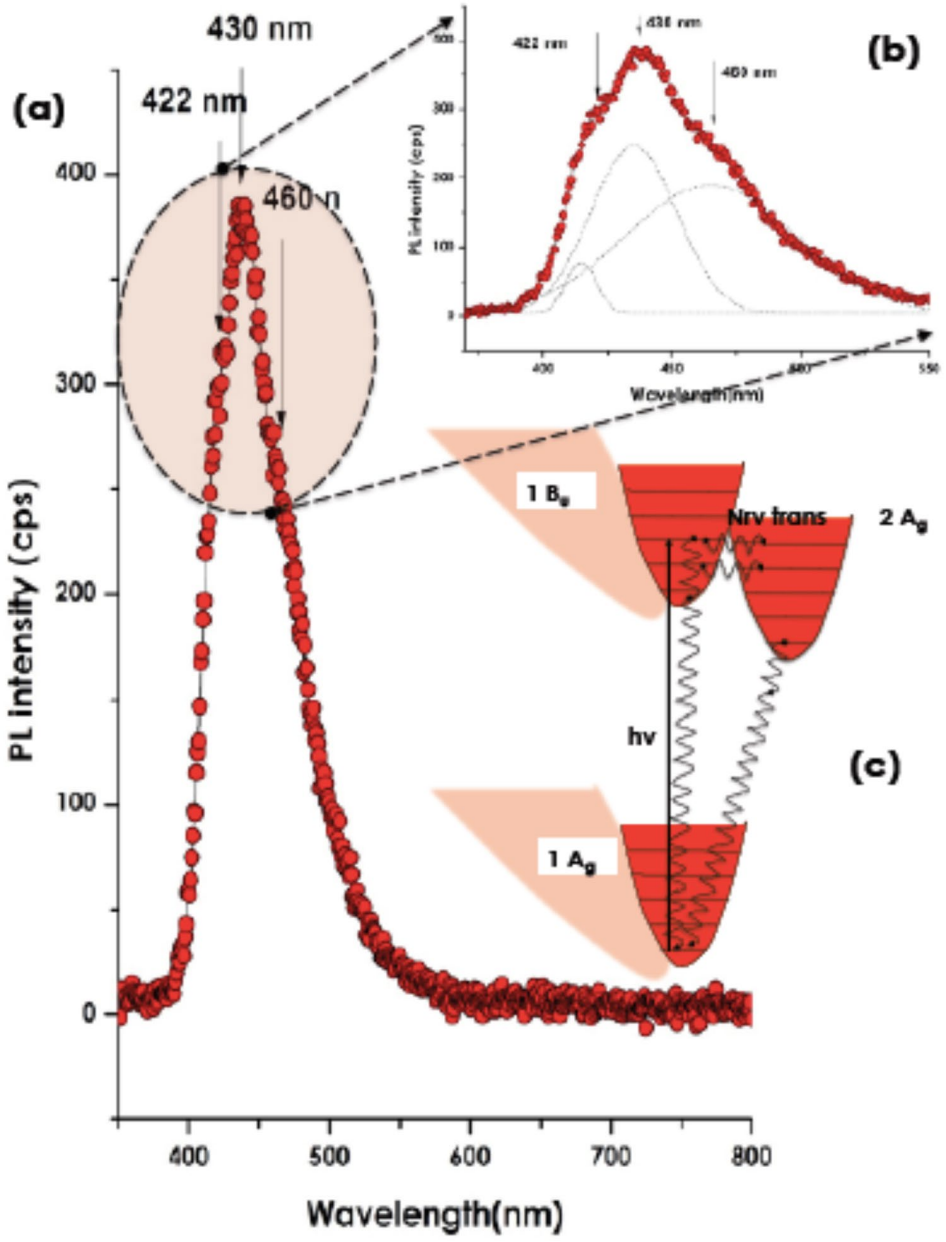
(**a**) Fluorescence emission of lycopene, (**b**) Zoom on the major Emission peaks and their convolution, (**c**) Schematic energy level diagram for carotenoids. Here 1A_g_, is the ground state and 1B_u_, the allowed excited state. 2A_g_, is a forbidden excited electronic state populated by non-radiative relaxation from the 1B_u_ state.

Figure 4a reports the fluorescence spectrum under a light emitting diode emitting at 240 nm. One observes a broad emission consisting in fact of the convolution of 3 emissions peaking at l_Max_, 422, 430 and 460 nm with width at half maximum Dl_1/2_ of 22.93, 40.64 and 72.94 nm respectively (Fig. 4b). Similar fluorescence patterns were observed by Fujii et al.^27–29^. According to Fuji et al.^27,28^, such a set of fluorescence emissions were attributed to ^1^Bu to 2Ag − 1Ag transitions via intermediary non radiative 1Bu to 2Ag transitions following an excitation from 1Ag to 1Bu as schematically summarized an inset of Fig. 4c.

**Figure 5:**
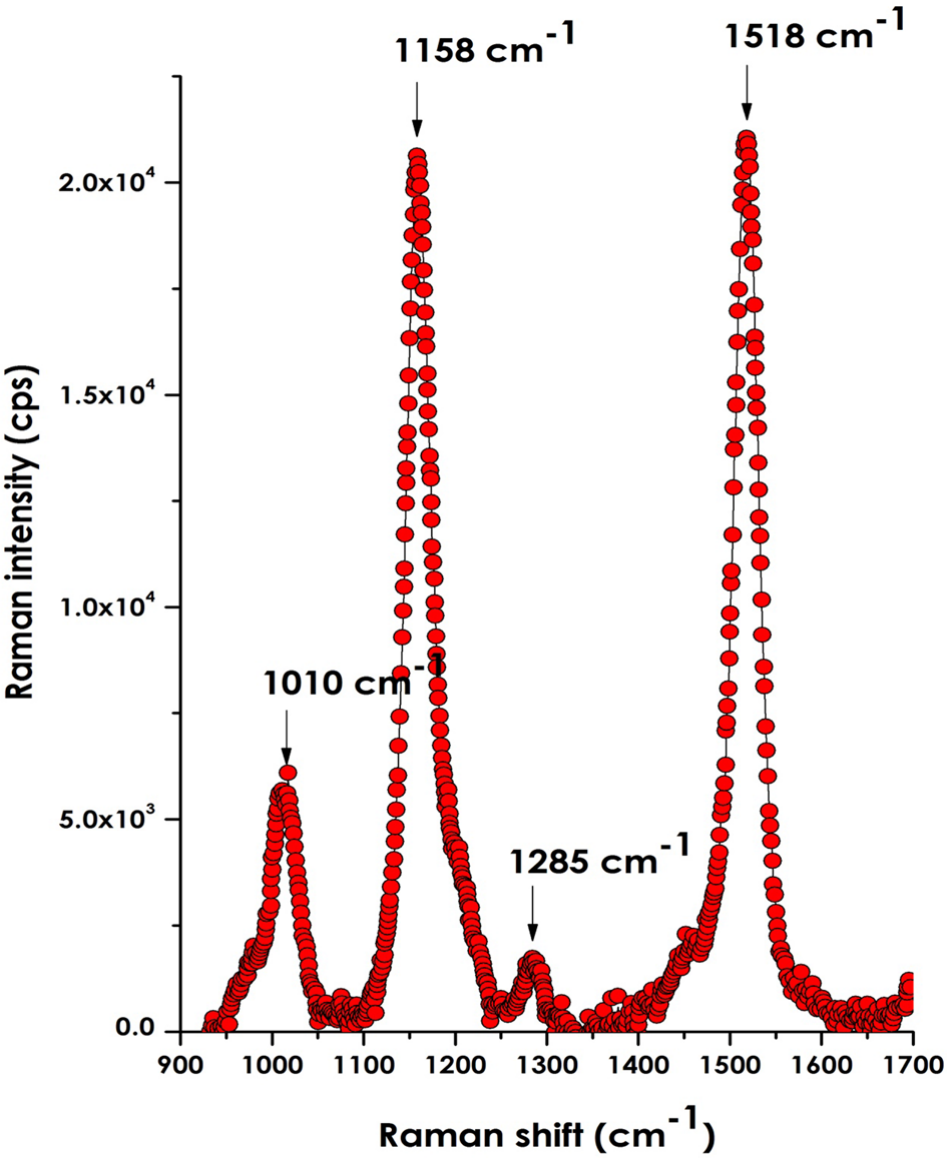
Raman spectrum under an argon laser excitation of 514.5 nm of Lycopene (this excitation wavelength coincides to resonance Raman conditions for Lycopene).

In view of checking the Lycopene’s quality & its purity, Raman spectroscopy studies were carried out on the extract. Figure 5 reports the Raman spectrum under an argon laser excitation of 514.5 nm. It happened that this special excitation wavelength coincides with the resonance Raman conditions for Lycopene^30,31^ and hence the Raman modes qre expected be intense, if any. Indeed, as one can observe, the Raman response is characterized by two prominent Stokes lines centered approximately at 1158, and 1518 cm^−1^, which have nearly similar relative intensities. Such emissions are intrinsic to Lycopene^30,31^, and, originate, from carbon–carbon single-bond and double-bond stretch vibrations of the conjugated backbone of the lycopene molecule. The relatively less intense emission peaking approximately at 1010 cm^−1^ is attributed to rocking motions of the molecule’s methyl components^30,31^. There is an additional vibrational mode located at 1285 cm^−1^ that was unable to be precisely identified.

### Z–scan method and measurement of third order nonlinear optical properties

**Figure 6:**
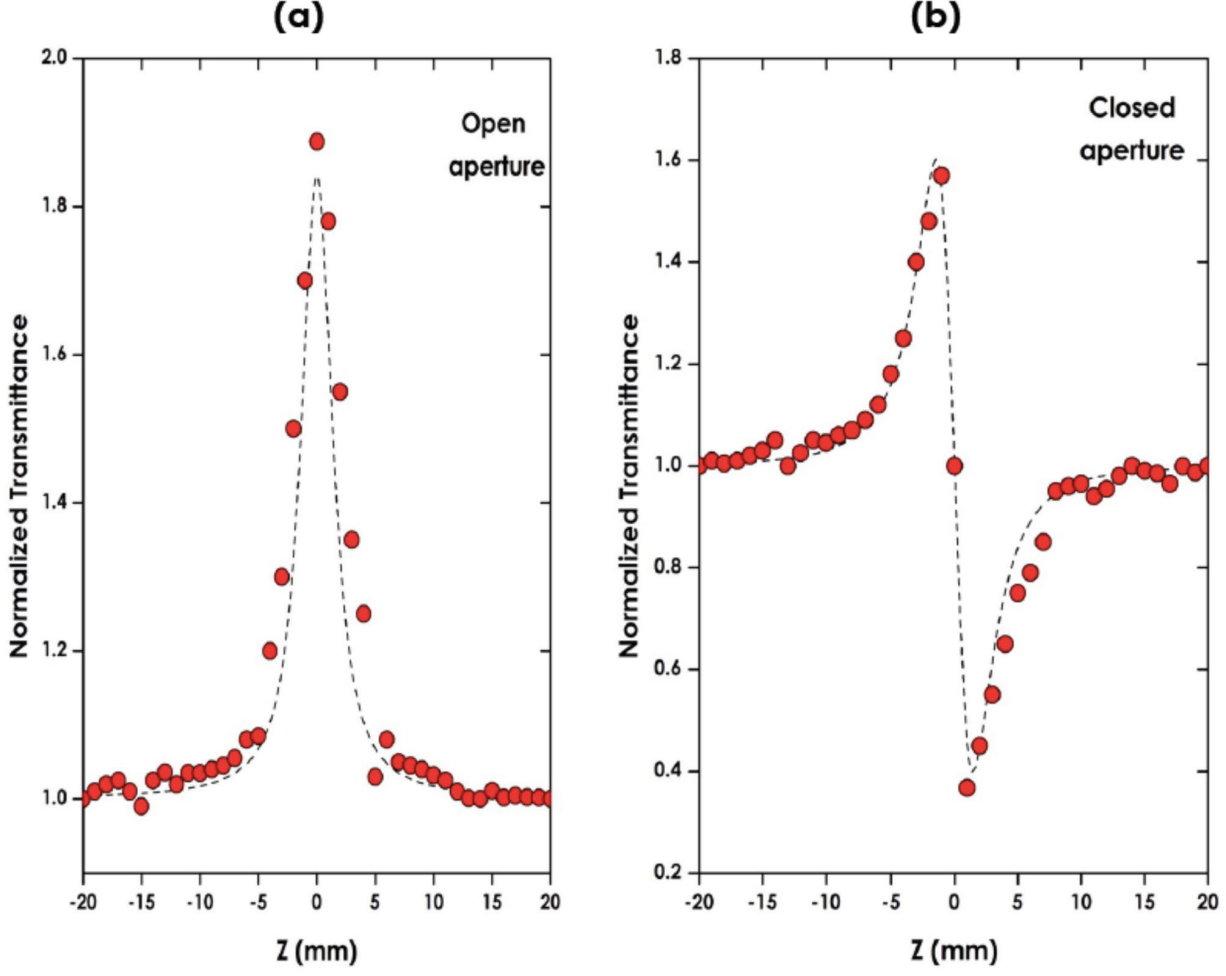
(**a**) The open aperture Z‒scan profile of lycopene, (**b**) The closed aperture Z–scan profile exhibiting a peak followed by a valley normalized transmittance indicating that the Lycopene exhibits self-defocusing behaviour and, a negative nonlinear index of refraction. The symbols are the experimental data. The solid lines are the best-fit curves calculated by the Z-scan theory.

The nonlinear index of refraction, *n*_*2*_ and nonlinear coefficient of absorption, *β* of lycopene are related to the real and imaginary factors of the third-order NLO susceptibility (*χ*^*(3)*^). Figure 6a illustrates the open aperture Z‒scan profile of *lycopene.* It is observed from such a figure that the transmitted intensity increases at the focus, and forms a fine peak which is an indication of a saturable absorption (SA). The solid line in Fig. 6a is the theoretical fit, which is relatively well-matched with the experimental profile. With the Saturable Absorption phenomenon as the driving NLO process in the investigated Lycopene sample, the transmittance of the natural pigment *Lycopene* is determined from the fitting curve of the open aperture is given by Sheik-Bahaenction^32^:

1 $$ T\left( z \right) = \mathop \sum \limits_{m = o}^{\infty } \frac{{ - q_{o}^{m} }}{{\left( {m + 1} \right)^{3/2} }} $$

2 $$ q_{o} \left( z \right) = \frac{{I_{o} L_{eff} \beta }}{{1 + \frac{{Z^{2} }}{{Z_{0}^{2} }}}} $$

3 $$ T\left( z \right) = 1 - \frac{{\left( {I_{o} L_{eff} \beta } \right)}}{{\left[ {2\sqrt {2\left( {1 + \frac{{Z^{2} }}{{Z_{0}^{2} }}} \right)} } \right]}} $$

where $L_{eff} = \left[ {1 - {\text{exp}}\left( { - \alpha_{o} L} \right)} \right]/\alpha_{o}$ is the effective length of the sample, L is the thickness of the sample, α_o_ is the linear absorption coefficient.

The closed aperture Z–scan measurements have been used to measure the magnitude and the sign of the nonlinear index of refraction of the sample. Figure 6b displays the pure nonlinear refraction curve of the Lycopene sample. A peak followed by a valley normalized transmittance curve is observed (Fig. 6b) indicating that the Lycopene exhibits a self-defocusing behaviour and, consequently, a negative nonlinear index of refraction. The observed self-defocusing effect in the natural pigment Lycopene is likely to be the result of a thermal nonlinearity. The normalized peak-valley difference ΔT_p–v_ as a function of on-axis phase shift ∣Δφ_0_∣ is given by:

4 $$ \Delta T_{P - V} = 0.406\left( {1 - S} \right)^{0.25} \Delta \emptyset_{o} $$

where $S = 1 - \exp \left( { - 2r_{0}^{2} /\omega_{0}^{2} } \right)$ is the linear aperture transmittance, ω_0_ is the beam radius and r_0_ denotes the aperture radius. The transmittance of the natural pigment is given by:

5 $$ T\left( z \right) = 1 + \Delta \emptyset_{o} \frac{4X}{{\left( {X^{2} + 1} \right)\left( {X^{2} + 9} \right)}} $$

where X = Z/Z_o_. The nonlinear index of refraction n_2_ of the sample is given by^32^:

6 $$ n_{2} = \frac{{\Delta \varphi_{0} \lambda }}{{2\pi I_{0} L_{eff} }}\left( {\frac{{cm^{2} }}{W}} \right) $$

where λ is the laser wavelength, and L_eff_ is the effective length of the sample. The real and imaginary components of third-order NLO susceptibility (χ^(3)^) are given by^32^:

7 $$ Re\left[ {\chi^{\left( 3 \right)} } \right]\left( {esu} \right) = \frac{{\varepsilon_{0} c^{2} n_{0}^{2} }}{{10^{4} \pi }}n_{2} \left( {\frac{{m^{2} }}{W}} \right) $$

8 $$ Im\left[ {\chi^{\left( 3 \right)} } \right]\left( {esu} \right) = \frac{{\varepsilon_{0} c^{2} n_{o}^{2} \lambda }}{{10^{2} 4\pi^{2} }}\beta \left( \frac{m}{W} \right) $$

where ε_0_ and c are the permittivity of the vacuum and the velocity of light. The calculated third-order NLO parameters of Lycopene (summarized in Table 1) are − 7.26 × 10^−12^ m^2^/W and − 0.20 × 10^−5^ m/W respectively. Therefore, the corresponding real and imaginary parts of the third-order NLO susceptibility [Re (χ^(3)^)] & [Im (χ^(3)^)] are − 2.45 × 10^−6^ and − 1.01 × 10^−6^ esu. Hence, the Lycopene third-order NLO susceptibility (χ^(3)^) is about 2.65 × 10^−6^ esu.

**Table 1:** The Third-order NLO parameters of *Lycopene.*

Experimental parameters	Obtained experimental values
Nonlinear index of refraction (*n*_*2*_)	− 7.26 × 10^−12^ m^2^/W
Nonlinear absorption coefficient (*β*)	− 0.20 × 10^−5^ m/W
Real part of third-order NLO susceptibility [Re (χ^(3)^)]	− 2.45 × 10^−6^ esu
Imaginary part of third-order NLO susceptibility [Im (χ^(3)^)]	− 1.01 × 10^−6^ esu
Third-order NLO susceptibility (χ^(3)^)	2.65 × 10^−6^ esu

It is to be pointed out that according to Fig. 3, Lycopene has a weak absorption at 650 nm yet the open-aperture Z-scan curve in Fig. 6a shows that Lycopene exhibits a saturable absorption (SA). What is the mechanism for this SA response?. It is to be highlighted that the saturable absorption is a nonlinear phenomenon which arises at high light input intensities at the focus. Hence, at sufficient light intensities at the focus, the ground state is excited into an upper energy state at such a rate there is insufficient time to decay back to the ground state before the ground state becomes depleted. The absorption cross-section of ground state is higher than that of excited state is also the mechanism of absorption saturation.

To sustain the thermal nature of the registered NLO response of the lycopene, on should highlight the following considerations. The laser beam, at the spot size was 1.15 mm. The measured Rayleigh length was 1.47 mm. Hence, the intensity at the focus was 1.047 kW/cm^2^. In lycopene pigment, the observed self-defocusing effect is the result of thermal nonlinearity as it is in most if not each and all organic dyes and pigments, thermal nonlinearity is the predominant. Under CW irradiation, the nonlinearity is due to thermal in nature and not because of other effects. This is confirmed from the following reasons; (i) the value of nonlinear refractive index n_2_ > 10^−5^ esu and (ii) A peak-valley separation of more than 1.7 times the Rayleigh range is the indication of thermal nonlinearity and indicates the observed nonlinear effect is the third-order process. Therefore, the observed nonlinearity in the pigment is due to thermal in nature.

Table 2 reports the NLO 3rd order susceptibility χ^(3)^ of various carotenoids and natural extracts published in the last decade. Also it displays the relative enhancement (χ^(3)^_Lyc_ − χ^(3)^_Pigm_)/χ^(3)^_Lyc_). This relative parameter, is a priori an optimal parameter as a comparative measure of χ^(3)^_Lyc_ relatively to that of a series of natural extracts reported in the scientific literature. As one can notice, it seems that the measured χ^(3)^_Lyc_ of Lycopene of 2.65 10^−6^ esu is, a priori, the highest value relatively to each & all natural compounds and carotenoids with conjugated π-electrons investigated so far. Indeed, the relative increase ((χ^(3)^_Lyc_ − χ^(3)^_Pigm_)/χ^(3)^_Lyc_) is within 42% and 93%.

**Table 2:** NLO 3rd order susceptibility χ^(3)^ of various carotenoids and natural extracts published in the last decade.

Pigment nature	Laser excitation (nm)	Absorption peak (nm)	Measurement Technique	χ^(3))^ (10^–6^ esu)	Relative enhancement (χ^(3)^_Lyc_ − χ^(3)^_Pigm_)/χ^(3)^_Lyc_	References
Beta- Carotene	532 1064	400–500	DFWM THG	0.87	67%	^11^
Violaxanthin	532 1064	400–500	DFWM THG	0.27	89.8%	^11^
Xanthophyll	532 1064	400–500	DFWM THG	0.19	93%	^11^
Chlorophyll	532 1064	400–500	DFWM THG	0.19	93%	^11^
Anthocyanin extracted from blueberry	635	629	Z-scan	0.528	80%	^33^
Anthraquinone dye (Acid green 25. Color Index: 61,570)	635	638	Z-scan	0.311	88%	^34^
Hisbiscus Rosa dye	532	347–515	Z-scan	0.577	78%	^8^
Bixa dye	532	494–500	Z-scan	0.577	78%	^9^
Chlorophyll-a Coriandrum Sativum	635	674	Z-scan	0.135	94%	^35^
Chlorophyll-a extracted from Andrographis paniculata	635	672	Z-scan	1.53	42%	^36^
β-carotenoid extracted from pyllanthus niruri	635	200–400	Z-scan	0.676	74%	^37^
Lycopene	650	483	Z-scan	2.65	Current study	Current

### Computational results: calculation of quantum molecular descriptors

The observed enhanced experimental value of the 3rd NLO susceptibility of Lycopene is likely to originate from a high electronic polarizability of the Lycopene and its 1-D π-electrons electronic conformation (Fig. 1). For such, it is necessary to investigate the Lycopene molecule’s LUMO and HOMO. Hence, the structure of Lycopene was optimized at B3LYP level of theory using the 6-311 basis set carried out using Gaussian 09^37^. At the same level of theory, frequency calculation was done on the optimized structure to make sure of the true minima. The orbital energies of HOMO and LUMO were calculated to obtain the quantum molecular descriptors.

**Figure 7:**
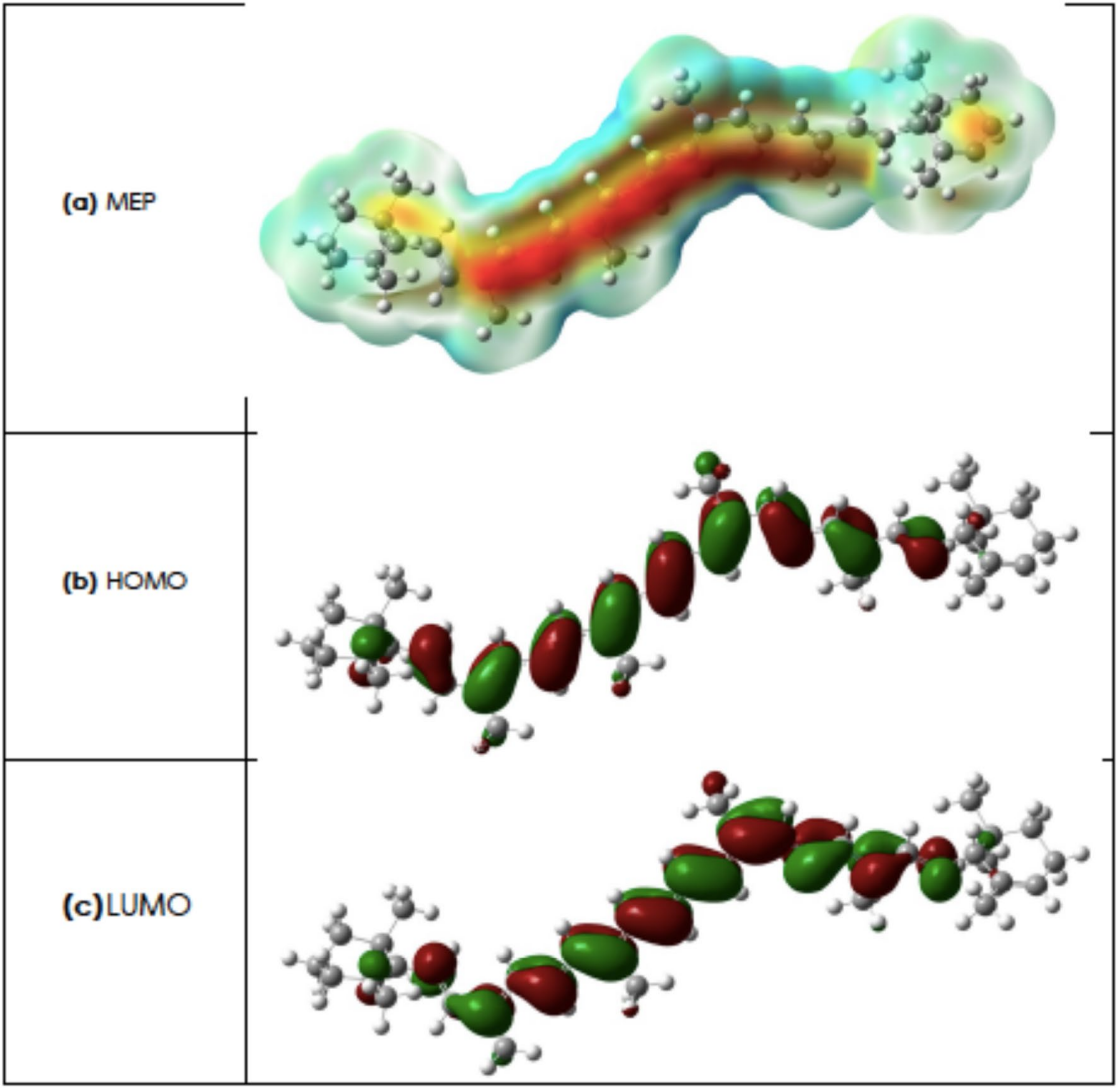
(**a**) Molecular Electrostatic Potential (MEP) of the optimized structure of Lycopene (the reddish regions indicate the most active sites of Lycopene, (**b**) The Highest Occupied Molecular Orbital (HOMO) and (**c**) the Lowest Unoccupied Molecular Orbital (LUMO), referred to as frontier orbitals.

The highest occupied molecular orbital (HOMO) and the lowest unoccupied molecular orbital (LUMO), referred to as frontier orbitals, play a significant role in chemical reactivity and molecular interactions^38^. The molecular electrostatic potential (MEP) and the HOMO–LUMO orbitals of the optimized structure of Lycopene are presented in Fig. 7. The reddish area in Fig. 7c indicates the most active sites of Lycopene. The derived energy of HOMO and LUMO orbital is − 4.62 and − 2.34 eV, respectively. The chemical potential (μ) can be used to assess the evasion affinity of a molecule from equilibrium. The chemical hardness (η) is a property that quantifies the charge transfer and chemical reactivity of a molecule while the Electronegativity ($\xi$) determines the ability of the molecule to attract electrons, and finally higher value of electrophilicity index (ω) means higher electrophilic power of the molecule^39,40^. The values of these parameters are reported in Table 3.

**Table 3:** The HOMO and LUMO energy, energy gap (eV), chemical potential (μ), chemical hardness (η), electronegativity ($\xi$), and electrophilicity (ω), (in eV) of Lycopene^39–41^.

$E_{HOMO}$	Energy of Highest Occupied Molecular Orbital	− 4.62 (eV)
$E_{LUMO}$	Energy of Lowest Unoccupied Molecular Orbital	− 2.34 (eV)
$E_{gap} = E_{LUMO} - E_{HOMO}$	Energy gap	2.28 (eV)
$\mu = \frac{{E_{LUMO} + E_{HOMO} }}{2}$	Chemical potential: used to assess the evasion affinity of a molecule from equilibrium	− 3.48 (eV)
$\eta = \frac{{E_{LUMO} - E_{HOMO} }}{2}$	Chemical hardness: a property that quantifies the charge transfer and chemical reactivity of a molecule	1.14 (eV)
$\chi = - \frac{{E_{LUMO} + E_{HOMO} }}{2}$	Electronegativity: determines the ability of a molecule to attract electrons	3.48 (eV)
$\omega = \frac{{\chi^{2} }}{2 \eta }$	Electrophilicity index: electrophilic power of the molecule	5.30 (eV)

The static dipole moment ($\mu_{x} ,\mu_{y} ,\mu_{z}$), and polarizabilities *(α*_*xx*_, *α*_*xy*_, *α*_*yy*_, *α*_*xz*_, *α*_*yz*_, *α*_*zz*_ and *β*_*xxx*_, *β*_*xxy*_, *β*_*xyy*_, *β*_*yyy*_, *β*_*xxz*_, *β*_*xyz*_, *β*_*yyz*_, *β*_*xzz*_, *β*_*yzz*_, *β*_*zzz*_) were calculated at the same level of theory analytically using the keyword Polar in Gaussian^41^ and reported in Tables 4, 5 and 6. The total static dipole moment $\left\langle \mu \right\rangle$, the mean polarizability $\left\langle \alpha \right\rangle$, the anisotropy of the polarizability $\Delta \alpha$ and the mean first hyperpolarizability $\left\langle {\beta { }} \right\rangle$ were calculated by utilizing the following equations

$$ \left\langle \mu \right\rangle = \sqrt {\mu_{x}^{2} + \mu_{y}^{2} + \mu_{z}^{2} } $$

$$ \left\langle \alpha \right\rangle = \frac{{\alpha_{xx} + \alpha_{yy} + \alpha_{zz} }}{3} $$

$$ {\Delta }\alpha = \sqrt {\frac{{\left( {\alpha_{xx} - \alpha_{yy} } \right)^{2} + \left( {\alpha_{yy} - \alpha_{zz} } \right)^{2} + \left( {\alpha_{zz} - \alpha_{xx} } \right)^{2} }}{2}} $$

$$ \beta_{x} = \beta_{xxx} + \beta_{xyy} + \beta_{xzz} $$

$$ \beta_{y} = \beta_{yyy} + \beta_{yxx} + \beta_{yzz} $$

$$ \beta_{z} = \beta_{zzz} + \beta_{zxx} + \beta_{zyy} $$

$$ \left\langle \beta \right\rangle = \sqrt {\beta_{x}^{2} + \beta_{y}^{2} + \beta_{z}^{2} } $$

**Table 4:** Static dipole moment of Lycopene calculated using DFT at B3LYP/6-311 level of theory(in a.u. unit).

$\mu_{x}$	$\mu_{y}$	$\mu_{z}$	$\mu$
0.193725	− 0.138999	0.115865	0.265094

**Table 5:** Static polarizability tensor of Lycopene calculated using DFT at B3LYP/6–311 level of theory in a.u. unit and converted values to esu.

Static polarizability	a.u	esu
$\alpha_{xx}$	1793.68000	265.8054 $\times { }10^{ - 24}$
$\alpha_{yy}$	456.82900	67.69749 $\times { }10^{ - 24}$
$\alpha_{zz}$	340.13700	50.40490 $\times { }10^{ - 24}$
$\alpha_{xy}$	− 105.76500	− 15.67332 $\times { }10^{ - 24}$
$\alpha_{xz}$	− 14.38610	− 2.131876 $\times { }10^{ - 24}$
$\alpha_{yz}$	− 33.10440	− 4.905741 $\times { }10^{ - 24}$
$\left\langle \alpha \right\rangle$	863.54867	127.9693 $\times { }10^{ - 24}$
$\Delta \alpha$	1412.18084	209.2711 $\times { }10^{ - 24}$

**Table 6:** Static first hyperpolarizability tensor of Lycopene calculated using DFT at B3LYP/6-311 level of theory (in a.u. unit) and converted values to esu.

Static hyperpolarizability	a.u	esu
$\beta_{xxx}$	11,543.00000	99.72252 $\times { }10^{ - 30}$
$\beta_{yyy}$	− 11.75330	− 0.1015393 $\times { }10^{ - 30}$
$\beta_{zzz}$	− 200.58700	− 1.732915 $\times { }10^{ - 30}$
$\beta_{xxy}$	− 1285.84000	− 11.10865 $\times { }10^{ - 30}$
$\beta_{xyy}$	198.00000	1.710566 $\times { }10^{ - 30}$
$\beta_{xxz}$	944.66600	8.161177 $\times { }10^{ - 30}$
$\beta_{xyz}$	3.08147	0.02662150 $\times { }10^{ - 30}$
$\beta_{yyz}$	72.36650	0.6251901 $\times { }10^{ - 30}$
$\beta_{xzz}$	− 82.92250	− 0.7163857 $\times { }10^{ - 30}$
$\beta_{yzz}$	− 90.53260	− 0.7821310 $\times { }10^{ - 30}$
$\beta_{x}$	11,658.07750	100.717E $\times { }10^{ - 30}$
$\beta_{y}$	− 1388.12590	− 11.9923E $\times { }10^{ - 30}$
$\beta_{z}$	816.44550	7.05345E $\times { }10^{ - 30}$
$\left\langle \beta \right\rangle$	11,768.78276	101.6731 $\times { }10^{ - 30}$

The values of polarizabilities and first-order hyperpolarizabilities are reported in atomic units (a.u.), which are converted into electrostatic units (esu) using conversion factor of $0.1482{ } \times { }10^{ - 24}$ esu for $\alpha$ and $8.6393{ } \times { }10^{ - 33}$ esu for β. The results in Table 5 indicate that the x component of hyperpolarizability tensor (along the main molecular axis) has a significant contribution on $\left\langle { \beta } \right\rangle$. The obtained values of static polarizability and first-order hyperpolarizability are $127.96{ } \times { }10^{ - 24}$ and $101.67{ } \times { }10^{ - 30}$ esu respectively. Since one of the most critical factors of the NLO system is the magnitude of molecular hyperpolarizability, the mean first hyperpolarizability of lycopene was compared with other organic molecules in Table 7, which shows significant NLO activity of lycopene. The substantial $\pi$ delocalization along the major molecular axis, as seen in Fig. 7, accounts for the relatively large hyperpolarizability of lycopene.

**Table 7:** Comparison of computed first order hyperpolarizability of lycopene with some other studied organic molecules.

Molecule	Structure	$\langle \beta \rangle$ $\times {10}^{-30}$ (esu)	Method/ref
Lycopene		101.6731	B3LYP/6-311 Current study
Lawsone		1.12	B3LYP/6-311 + G (d) ^41^
1,4-Naphthoquinone		2.85	B3LYP/6-311 + G (d) ^41^
Juglone		5.22	B3LYP/6-311 + G (d) ^41^
7-Nitro-9H-fluoren-2-ylamine		30.20	HF/6-31G ^42^
[2-[7-Nitro-9H-fluoren-2-yl)-vinyl]-1-1’-dipyrrolidine		83.18	HF/6-31G ^42^
[2-[7-(2,2-Dinitro-vinyl)-9H-fluoren-2-yl]-vinyl]-1,1’-dipyrrolidine		209.44	HF/6-31G ^42^
2,2′-(quinoline- 2,4-diyl)bis(9-methyl-9H-carbazole		28.31	HF/6-31G ^43^
(E)-2-cyano-3-(5′-(9-decyl-7-(4-(9-decyl-9H-carbazol -2-yl)quinolin-2-yl)-9H-carbazol-3-yl)-[2,2′-bithiophen]-5-yl)acrylic acid		433.313	HF/6-31G ^43^

The above derived values of the static polarizability ($\Delta \alpha =$ 209.2711 $\times { }10^{ - 24}$ esu) & hyperpolarizabitlity ($\left\langle \beta \right\rangle = 101.6731{ } \times { }10^{ - 30}$) of Lycopene , seem pointing to a higher electronic polarizability of the Lycopene molecule corroborating therefore the experimentally observed enhanced third-order NLO response χ^(3)^.

## Conclusion

Linear & NLO properties of Lycopene were investigated. While the Raman & the UV–VIS studies confirmed the significant purity of the studied Lycopene, their nonlinear optical investigations by Z-scan indicated that Lycopene exhibits a significant optical nonlinearity response of a third order in nature under a CW laser excitation. Its corresponding third-order NLO susceptibility (χ^(3)^) was found to be substantially elevated reaching the high value of 2.65 X 10^−6^ esu. According to the published literature, such a value is likely to be the highest registered NLO susceptibility of any natural extract including Beta-Carotene, Violaxanthin, Xanthophyll, and Chlorophyll. More accurately, the relative increase ((χ^(3)^_Lyc_ − χ^(3)^_Pigm_)/χ^(3)^_Lyc_) lies within the range of 42%–93%. As a follow up of this study, NLO studies on Lycopene embedded in various optically passive or active polymeric membranes as well as in form of spin-coated nanocomposite thin films. This study reveals that natural Lycopene is a promising material for third order nonlinear optical devices application.

### Statement

It is to be mentioned that:

(i) All methods were carried out in accordance with relevant guidelines and regulations of international standards.

(ii) The authors are ready, upon request, to share their raw data, either by providing it in a supplementary file or depositing it in a public repository and providing the details on how to access.
